# Contribution of sleep quality to fatigue following a stroke: a cross-sectional study

**DOI:** 10.1186/s12883-021-02174-z

**Published:** 2021-04-07

**Authors:** Lily Yuen Wah Ho, Claudia Kam Yuk Lai, Shamay Sheung Mei Ng

**Affiliations:** 1 School of Nursing, The Hong Kong Polytechnic University, Hung Hom, Kowloon Hong Kong SAR; 2 Department of Rehabilitation Sciences, The Hong Kong Polytechnic University, Hung Hom, Kowloon Hong Kong SAR

**Keywords:** Fatigue, Independent living, Regression analysis, Sleep, Stroke

## Abstract

**Background:**

The prevalence of fatigue and sleep disturbances is high in stroke populations. Sleep quality can be targeted by interventions to alleviate fatigue following a stroke. This study aimed to determine the prevalence of fatigue and poor sleep quality, and to quantify the contribution of sleep quality to fatigue following a stroke, in chronic (≥1 year) stroke survivors.

**Methods:**

A cross-sectional design was adopted. A total of 112 stroke survivors (mean age ± standard deviation [year], 64.18 ± 5.77) at 6.08 ± 4.80 years post-stroke completed this study. All participants were assessed using the Fatigue Assessment Scale, the Pittsburgh Sleep Quality Index, the Visual Analogue Scale-Pain, the Fugl-Meyer Assessment of the upper and lower extremities, the 5-Time Sit-To-Stand Test, the Epworth Sleepiness Scale, the Frenchay Activities Index, the Life-Space Assessment, the Community Integration Measure, and the Multidimensional Scale of Perceived Social Support. Pearson and partial correlation coefficients were used to examine the correlation between fatigue and other variables. A multiple linear regression (the forced entry method) was performed to quantify the independent contribution of sleep quality to prediction of fatigue.

**Results:**

Of the 112 participants, 52.7% reported experiencing fatigue and 64.3% reported poor sleep quality. Sleep quality could independently account for 5.9% of the variance in fatigue scores after a stroke.

**Conclusions:**

There is a high prevalence of fatigue and poor sleep quality in Chinese stroke survivors. Sleep quality is an independent predictor of fatigue in those living in the community who have survived a stroke for a year or longer.

## Background

Stroke survivors often experience fatigue, which is described as physical or mental tiredness [1]. Its prevalence was found to be 50% in a systematic review and meta-analysis involving 49 studies on stroke survivors [2]. Fatigue following a stroke can lead to a significant reduction in participation in stroke rehabilitation [3] and in the activities of daily living [4]. Fatigue can also affect the daily roles and occupational performance of stroke survivors [5], and therefore hinder their integration into the community. Fatigue following a stroke has been found to be significantly negatively correlated with time spent stepping per day and daily step counts [6]. Fatigue may also lower an individual’s motivation to reach out, thus limiting that person’s life-space, which is the physical extent of the environment in which a person moves in daily life [7].

Fatigue in stroke survivors can be related to various factors. For example, fatigue following a stroke has been found to relate to demographic characteristics such as age [4] and female gender [8]. It is also associated with physical factors, such as pain [9] and self-perceived strength [10], although the relationship between fatigue and objective measurements of muscle strength is unclear. Stroke survivors with motor impairments need to make greater efforts to perform a movement, and this extra effort can cause tiredness [11]. Weakness in the paretic muscles after stroke also leads to a feeling of fatigue and to a decline in the performance of motor tasks. However, fatigue can be mediated by social support [12].

Literature reviews suggest that sleep disturbances and sleep problems coexist with fatigue after a stroke [13, 14]. A review of fatigue in neurological diseases showed that poor sleep is a predisposing factor of fatigue [15]. Sleep disorders have been reported by 78% of stroke survivors [16]. Poor sleep quality can lead to daytime sleepiness after a stroke.

Fatigue is correlated with several subjective measurements of sleep. Fatigue has been shown to be significantly associated with sleep quality [17, 18], increased daytime sleepiness [6, 17], the duration of daytime naps, the frequency of waking after the onset of sleep, the frequency of daytime naps [17], and sleep disturbances [9, 19, 20]. However, these cross-sectional and longitudinal studies have investigated the relationships between fatigue and sleep variables within a year after a stroke [6, 9, 17–20]. Only one study extended the post-stroke duration to 15 months, and showed that Fatigue Severity Scale results were significantly correlated with those from the sleep section of the Nottingham Health Profile [21]. Despite the use of various sleep parameters in studies of fatigue, sleep quality, which takes both subjective and objective sleep parameters into consideration [22], has not been widely studied. In addition, as noted above, the correlation between fatigue and sleep has mostly been studied only within a year after a stroke. Although Wu et al. [13] argued that the mechanisms of fatigue might not be the same at different stages of recovery from a stroke, few studies have explored the relationship between fatigue and sleep quality after more than a year after a stroke. This knowledge gap needs to be addressed.

Sleep quality has been reported to be a significant predictor of fatigue in people with multiple sclerosis [23] and epilepsy [24], but its power to predict fatigue following a stroke is unclear. As sleep quality can be improved through physical interventions such as exercise training in older populations [25], it may be a potential target component in interventions to alleviate fatigue following a stroke. Therefore, the objectives of this study were (1) to determine the prevalence of fatigue and poor sleep quality in community-dwelling people who had been diagnosed with stroke a year or more ago, and (2) to quantify the relative contribution of sleep quality to fatigue following a stroke.

## Methods

### Design, setting, and sampling

This was a cross-sectional study. A convenience sample of stroke survivors was recruited from self-help groups in the Hong Kong Special Administrative Region from January to August 2019. The criteria for inclusion in this study were as follows: (1) aged ≥55; (2) having had a confirmed diagnosis of stroke ≥1 year previously; and (3) residing and ambulatory in the community. People who were on drug trials, had other neurological diseases, experienced transient ischemic attacks or unstable medical conditions, or who were unable to give their informed consent were excluded. The sample size was estimated based on a rule-of-thumb approach, namely, ≥50 + 8 *m*, where *m* is the number of predictors [26]. Based on the inclusion of up to eight variables in the regression analysis, it was determined that 114 participants would be needed.

### Measures

In selecting the scales, consideration was given to whether the scales could capture the corresponding constructs in stroke populations, whether they are commonly used in studies of stroke populations, and whether they were specifically designed for stroke populations.

The Chinese version of the Fatigue Assessment Scale (C-FAS) [27], which captures the physical and mental fatigue described by stroke survivors [1], was used to assess the level of fatigue. The first five items of the C-FAS assess physical fatigue and the second five items assess mental fatigue. Total scores range from 10 to 50. A score of ≥22 indicates fatigue [28]. The C-FAS was found to have good internal consistency (Cronbach’s α = 0.82) and test–retest reliability (intraclass correlation coefficient [ICC] = 0.92) in stroke populations [27].

The Cantonese version of the Pittsburgh Sleep Quality Index (CPSQI), which assesses nighttime sleep quality in the past month [29], was used. It consists of 19 items, with the total score ranging from 0 to 21, where a score of > 5 indicates poor sleep quality [30]. The internal consistency was found to be good (Cronbach’s α = 0.75), without a significant difference over a 1-week period, in a Chinese population [29].

The Visual Analogue Scale-Pain (VAS-Pain) was used to assess the level of pain during the assessment. Respondents select a specific point along a 10-cm-long line that best represents their pain level (“0” = no pain; “10” = worst pain). Both the inter-rater reliability (ICC = 0.79) and the intra-rater reliability (ICC = 0.70) are satisfactory in stroke survivors [31].

The Fugl-Meyer Assessment of the upper (FMA-UE) and lower extremities (FMA-LE) [32], which was designed specifically for stroke populations, was used to assess motor control of paretic limbs. The FMA-UE contains 33 items, with total scores ranging from 0 to 66; the FMA-LE contains 17 items, with total scores ranging from 0 to 34. Higher scores represent better motor capacity [32]. The test–retest reliability of the FMA-UE over 7 days (ICC = 0.97) [33] and that of the FMA-LE over 5–10 days (ICC = 0.94) were found to be excellent in stroke survivors [34].

The Five-Time Sit-To-Stand (FTSTS) Test [35] was used to assess the stroke survivors’ functional muscle strength in their lower limbs. Functional muscle strength reflects the force-producing capability of a muscle. Both the intra-rater reliability and test–retest reliability of the FTSTS Test are excellent (ICC > 0.97) in people with stroke [35].

The Chinese version of the Epworth Sleepiness Scale (CESS) was used [36], which is the scale most commonly used to assess daytime sleepiness in stroke populations [37]. It consists of eight items, where the total scores range from 0 to 24, with higher scores representing higher levels of sleepiness. In stroke survivors, the Epworth Sleepiness Scale shows a good fit with the Rasch model, with a nonsignificant overall chi-square probability (χ^2^ = 30.4, *p* = 0.173), and the scale is unidimensional [38].

The Chinese version of the Frenchay Activities Index (CFAI) [39] was used to assess the frequency of participation in activities as recommended by the American Heart Association’s Classification of Stroke Outcome Task Force [40]. The CFAI consists of 15 items, with total scores ranging from 0 to 45. Higher scores represent more frequent participation [39]. The test–retest reliability was found to be good (ICC = 0.89) in survivors of stroke in Taiwan [41].

The Life-Space Assessment, which considers the environmental and personal factors affecting mobility, was used to measure the respondents’ life-space along five levels. The total score ranges from 0 to 120, where a higher score represents a greater life-space. The instrument has excellent test–retest reliability (ICC = 0.95–0.97) [42]. In stroke survivors, the test–retest reliability in terms of the kappa statistic was found to be 0.99 [43]. A Cantonese version, which was translated from the original English version [42] based on Beaton et al.’s guidelines [44], was adopted in this study. Prior to this study, the internal consistency and the test–retest reliability of the Cantonese version of the Life-Space Assessment (CLSA) over 7–10 days were examined. Both were found to be satisfactory (Cronbach’s α = 0.73, ICC = 0.95).

The Cantonese version of the Community Integration Measure (CIM-C) [45] was used to measure community integration. The CIM-C is consistent with the activities and participation sections of the International Classification of Functioning, Disability, and Health framework. The CIM-C consists of 10 items, with total scores ranging from 10 to 50. Higher scores represent better community integration. The internal consistency (Cronbach’s α = 0.84) and the test–retest reliability (ICC = 0.84) of the CIM-C were found to be good in stroke populations [45].

The Chinese version of the Multidimensional Scale of Perceived Social Support (MSPSS-C) [46] was used to assess social support, as it addresses the subjective feeling of social support. The MSPSS-C consists of 12 items. The total score ranges from 12 to 84, with a higher score indicating a higher level of perceived social support. The internal consistency of the original scale was found to be good (Cronbach’s α = 0.88) and the test–retest reliability was found to be 0.85 over 2–3 months [47]. The MSPSS-C was found to have good internal consistency (Cronbach’s α = 0.95) in family caregivers of stroke survivors [48].

### Procedures

This study was approved by the ethics committee of the authors’ university and conducted in accordance with the Declaration of Helsinki. The objectives and procedures of the study were explained to the participants before they were asked to sign a form giving their written informed consent to participate. Self-reported socio-demographic data were collected and clinical outcomes were measured using the abovementioned scales.

### Statistical analysis

The Statistical Package for the Social Sciences (version 25) was used to analyze the data. Socio-demographic data and clinical outcomes were summarized and the prevalence of fatigue and poor sleep quality among the participants was determined by descriptive statistics. A combination of the visual inspection of histograms and normal Q-Q plots, the Kolmogorov–Smirnov test of normality, and *Z*-tests using skewness and kurtosis showed that the data did not significantly deviate from normality.

Pearson correlation coefficients were used to examine the correlation between C-FAS scores and other variables. The partial correlation coefficients between C-FAS scores and other variables were examined after controlling for CESS scores and FTSTS time to eliminate their effects on other variables. The CESS score was used as a control variable because sleepiness can be a sign of fatigue [49]. The FTSTS time was also used as a control variable because self-perceived strength is associated with fatigue [10].

A multiple linear regression (the forced entry method) was used because this study was confirmatory in nature. The aim was to quantify the independent contribution of sleep quality to C-FAS scores. Multicollinearity was tested by calculating the variance inflation factors. All variance inflation factors were less than 1.9. These values were acceptable, as they indicated that there were no strong linear relationships between the predictors. Confounding variables, including the CESS scores and FTSTS time, were adjusted for. Significant correlates were entered into the regression model. The variable with the weakest correlation with C-FAS scores was entered into the model first. Sleep quality was entered last, to quantify the independent contribution of sleep quality to predicting C-FAS scores. Participants with missing data were excluded from the analysis. A *p*-value of less than 0.05 was considered statistically significant.

## Results

### Characteristics of the participants

A total of 115 stroke survivors participated in this study, three of whom failed to complete the entire assessment battery, which left 112 participants. A summary of their socio-demographic data and clinical outcomes is given in Table 1. Of the participants, 52.7% reported experiencing fatigue and 64.3% reported poor sleep quality, as measured by the CPSQI.

**Table 1:** Socio-demographic characteristics and clinical outcomes of the participants (*N* = 112)

	Mean ± Standard deviation
Age	64.18 ± 5.77
Time since last stroke (years)	6.08 ± 4.80
C-FAS sum score	22.72 ± 6.19
C-FAS physical score	12.42 ± 3.90
C-FAS mental score	10.41 ± 3.30
CPSQI	7.51 ± 3.85
VAS-Pain	2.27 ± 2.61
FMA-UE	41.87 ± 20.74
FMA-LE	22.87 ± 5.64
FTSTS Test (seconds)	17.58 ± 7.80
CESS	7.06 ± 4.54
CFAI	21.21 ± 7.43
CLSA	70.46 ± 18.45
CIM-C	43.15 ± 6.15
MSPSS-C	59.53 ± 16.88
	**N (%)**
Gender	
Male	74 (66.1)
Female	38 (33.9)
Use of walking aids	
Yes	86 (76.8)
No	26 (23.2)
Hemiplegia on the dominant hand	
Yes	57 (50.9)
No	55 (49.1)

*CESS* Chinese version of the Epworth Sleepiness Scale, *CFAI* Chinese version of the Frenchay Activities Index, *C-FAS* Chinese version of the Fatigue Assessment Scale, *CIM-C* Cantonese version of the Community Integration Measure, *CLSA* Cantonese version of the Life-Space Assessment, *CPSQI* Cantonese version of the Pittsburgh Sleep Quality Index, *FMA-LE* Fugl-Meyer Assessment of the lower extremities, *FMA-UE* Fugl-Meyer Assessment of the upper extremities, *FTSTS* 5-Time Sit-To-Stand, *MSPSS-C* Chinese version of the Multidimensional Scale of Perceived Social Support, *VAS-Pain* Visual Analogue Scale-Pain

### Relationships between C-FAS scores and other variables

Significant correlations were found between the scores of the C-FAS and those of the CPSQI (*r* = 0.306, *p* = 0.001), FMA-UE (*r* = 0.191, *p* = 0.043), FMA-LE (*r* = 0.195, *p* = 0.040), CESS (*r* = 0.370, *p* < 0.001), CFAI (*r* = − 0.279, *p* = 0.003), CIM-C (*r* = − 0.243, *p* = 0.010), and MSPSS-C (*r* = − 0.223, *p* = 0.018). There was no correlation of the C-FAS scores with age, gender, use of walking aids, time since the last stroke, pain, functional muscle strength, or life-space (Table 2).

**Table 2:** Pearson correlation coefficients between the C-FAS and other variables

Variables	Pearson correlation coefficients with C-FAS scores	*P*
Age	0.042	0.658
Gender	−0.084	0.378
Use of walking aids	−0.056	0.561
Time since last stroke	− 0.078	0.415
CPSQI	0.306	0.001^†^
VAS-Pain	−0.029	0.765
FMA-UE	0.191	0.043*
FMA-LE	0.195	0.040*
FTSTS Test	0.105	0.270
CESS	0.370	< 0.001^†^
CFAI	−0.279	0.003^†^
CLSA	−0.092	0.335
CIM-C	−0.243	0.010*
MSPSS-C	−0.223	0.018*

*CESS* Chinese version of the Epworth Sleepiness Scale, *CFAI* Chinese version of the Frenchay Activities Index, *C-FAS* Chinese version of the Fatigue Assessment Scale, *CIM-C* Cantonese version of the Community Integration Measure, *CLSA* Cantonese version of the Life-Space Assessment, *CPSQI* Cantonese version of the Pittsburgh Sleep Quality Index, *FMA-LE* Fugl-Meyer Assessment of the lower extremities, *FMA-UE* Fugl-Meyer Assessment of the upper extremities, *FTSTS* 5-Time Sit-To-Stand, *MSPSS-C* Chinese version of the Multidimensional Scale of Perceived Social Support, *VAS-Pain* Visual Analogue Scale-Pain

*p < 0.05

^†^*p* < 0.01

After controlling for the FTSTS time and CESS scores, significant partial correlations were found between the scores of the C-FAS and those of the CPSQI (*r* = 0.265, *p* = 0.005), CFAI (*r* = − 0.251, *p* = 0.008), CIM-C (*r* = − 0.216, *p* = 0.023), and MSPSS-C (*r* = − 0.230, *p* = 0.016). The correlations between the C-FAS scores and those of the FMA-UE and FMA-LE became insignificant (Table 3).

**Table 3:** Partial correlation coefficients (controlling for the FTSTS time and CESS scores) between the C-FAS and other variables

Variables	Partial correlation coefficients with C-FAS scores	*P*
CPSQI	0.265	0.005^†^
FMA-UE	0.179	0.062
FMA-LE	0.159	0.096
CFAI	−0.251	0.008^†^
CIM-C	−0.216	0.023*
MSPSS-C	−0.230	0.016*

*CFAI* Chinese version of the Frenchay Activities Index, *C-FAS* Chinese version of the Fatigue Assessment Scale, *CIM-C* Cantonese version of the Community Integration Measure, *CPSQI* Cantonese version of the Pittsburgh Sleep Quality Index, *FMA-LE* Fugl-Meyer Assessment of the lower extremities, *FMA-UE* Fugl-Meyer Assessment of the upper extremities, *MSPSS-C* Chinese version of the Multidimensional Scale of Perceived Social Support

*p < 0.05

^†^*p* < 0.01

### Multiple linear regression

The entire model, including the FTSTS time and the scores of the CESS, FMA-UE, FMA-LE, MSPSS-C, CIM-C, CFAI, and CPSQI, accounted for 32.2% of the variance in the C-FAS scores (*F* [8, 103] = 7.575, *p* < 0.001). After adjusting for the FTSTS time and CESS scores, the CPSQI scores independently accounted for 5.9% of the variance in the C-FAS scores, and the predictive power of the model improved significantly (*F*-change 9.667, *p* = 0.002) (Table 4).

**Table 4:** Multiple linear regression (forced entry) relating the C-FAS with other variables

Independent variables	R^2^ (Adjusted R^2^)	R^2^ change	B (S.E.) (unstandardized coefficient)	β(standardized coefficient)	*P*
**Model 1**	0.154 (0.138)	0.154			
FTSTS Test			0.103 (0.070)	0.130	0.144
CESS			0.518 (0.121)	0.379	< 0.001^†^
**Model 2**	0.185 (0.155)	0.031			
FTSTS Test			0.140 (0.072)	0.177	0.054
CESS			0.467 (0.123)	0.342	< 0.001^†^
FMA-UE			0.037 (0.033)	0.124	0.264
FMA-LE			0.090 (0.125)	0.082	0.470
**Model 3**	0.311 (0.265)	0.126			
FTSTS Test			0.039 (0.073)	0.050	0.589
CESS			0.406 (0.116)	0.297	0.001^†^
FMA-UE			0.064 (0.032)	0.213	0.051
FMA-LE			0.108 (0.119)	0.098	0.365
MSPSS-C			−0.062 (0.033)	−0.168	0.066
CIM-C			−0.082 (0.092)	−0.082	0.373
CFAI			−0.235 (0.081)	−0.282	0.005^†^
**Model 4**	0.370 (0.322)	0.059			
FTSTS Test			0.026 (0.070)	0.032	0.714
CESS			0.361 (0.112)	0.265	0.002^†^
FMA-UE			0.084 (0.032)	0.282	0.009^†^
FMA-LE			0.058 (0.115)	0.053	0.615
MSPSS-C			−0.071 (0.032)	−0.193	0.030*
CIM-C			−0.032 (0.090)	−0.032	0.721
CFAI			−0.226 (0.078)	−0.271	0.005^†^
CPSQI			0.413 (0.133)	0.257	0.002^†^

*CESS* Chinese version of the Epworth Sleepiness Scale, *CFAI* Chinese version of the Frenchay Activities Index, *CIM-C* Cantonese version of the Community Integration Measure, *CPSQI* Cantonese version of the Pittsburgh Sleep Quality Index, *FMA-LE* Fugl-Meyer Assessment of the lower extremities, *FMA-UE* Fugl-Meyer Assessment of the upper extremities, *FTSTS* 5-Time Sit-To-Stand, *MSPSS-C* Chinese version of the Multidimensional Scale of Perceived Social Support

*p < 0.05

^†^*p* < 0.01

## Discussion

This study showed, for the first time, the prevalence of poor sleep quality and the contribution of sleep quality to fatigue in chronic stroke survivors. The findings highlight the importance of improving sleep quality to alleviate fatigue in this population.

### Prevalence of fatigue

The prevalence of fatigue in this study (52.7%) was consistently higher than that in previous studies conducted over 6 years after a stroke (37–48%) [10, 50, 51]. This may be because our participants were active members of self-help groups, and thus have actively taken part in social activities held by the groups, resulting in a higher prevalence of fatigue. Another possible reason is studies’ use of different fatigue scales, with varying classifications of fatigue. Notably, stroke survivors with depressive symptoms are at a significantly higher risk of experiencing fatigue (adjusted odds ratio = 8.86) [52]. Fatigue is also significantly correlated with anxiety and cognitive functioning in stroke survivors [53]. However, these possible contributors to the increased prevalence of fatigue were not examined in this study.

### Prevalence of poor sleep quality

The prevalence of poor sleep quality in this study (64.3%) was lower than that at the acute stage of stroke, but higher than that at the rehabilitation stage. Within 2 weeks after a stroke, 71.3% of stroke survivors in a study by Kim et al. had experienced poor sleep quality [54]. Our participants were chronic stroke survivors who were living at home, but Kim et al.’s participants were still in the acute stage and living in a hospital [54]. The medical care provided in the hospital in Kim et al.’s study [54], such as the frequent monitoring of vital signs, might have disturbed the participants’ sleep, resulting in a higher prevalence of fatigue in this group than in our participants. In addition, sleep-disordered breathing, which can disturb the sleep of stroke survivors, is most severe at the acute stage [55]. In a study conducted in a rehabilitation setting, only 39.1% of stroke in-patients experienced poor sleep quality [56]. Exercise training in that rehabilitation setting might have improved the quality of the in-patients’ sleep, as exercise promotes the consumption of energy and leads to an increased secretion of endorphins [25]. In contrast, our participants had been discharged from all rehabilitation services and were no longer receiving rehabilitation exercise training. Furthermore, those with sleep disorders might have received sleep medications in a rehabilitation setting. Yet healthcare providers for community-dwelling stroke survivors, as in our study, might not be as observant of such problems as they would be with in-patients. As identified by an item in the CPSQI, only 8.9% of our participants took medications to help them sleep. This might have resulted in a higher prevalence of poor sleep quality in this study than in rehabilitation settings.

### Sleep quality predicts levels of fatigue

Consistent with previous studies conducted within a year post-stroke [6, 9, 17–20], this study identified a significant and positive correlation between fatigue and sleep quality. The regression analysis also provided evidence for the contribution of sleep quality to fatigue at a year or more after a stroke. Sleep quality was a significant predictor of fatigue scores in this study, but not in other studies assessing sleeping quality and sleep disturbances conducted within 1–2 weeks after a stroke [17–19]. At the acute stage, biological triggers such as inflammatory biomarkers and neuroendocrine changes may play more important roles than sleep quality [13] and outweigh its effects. Over time, however, other factors such as sleep quality may continue to contribute to fatigue in chronic stroke survivors. This may explain the greater ability of sleep quality to explain fatigue scores in this study.

In stroke survivors, sleep quality may contribute to fatigue through changes in a person’s brain activity and in the macrostructure and microstructure of that person’s sleep [57]. For example, the lesions caused by a stroke may change an individual’s sleep–wake cycle and sleep circadian rhythm, and affect different stages of sleep. These changes can lead to various sleep disorders such as insomnia, which in turn can result in fatigue [57]. Conversely, the increased levels of fatigue in stroke survivors can in turn disturb their sleep–rest patterns [5]. Sleep helps to restore a person’s physical energy, and also has positive effects on mental performance such as memory consolidation [58]. Further studies are warranted to investigate the complex relationship between fatigue and sleep quality.

The variance in fatigue scores explained by sleep quality, as an independent predictor, was 5.9% in this study. This was slightly lower than that explained by pain (7%), but higher than that explained by depression (4%) and anxiety (4%) in a previous cross-sectional study that investigated psychological distress and fatigue in 98 stroke survivors [9]. In studies of people with other neurological diseases, sleep quality accounted for 11% of the variance in the fatigue scores of people with epilepsy [24] and 25% of the variance in those of people with multiple sclerosis [23]. Other than the use of different fatigue scales, different types of regression, and the differences in the pathophysiologies of fatigue in stroke, epilepsy, and multiple sclerosis patients, another possible explanation for the low variance in the fatigue scores explained by sleep quality in this study is that fatigue following a stroke is multifactorial [13]. Consistent with that possibility, in this study fatigue was found to correlate with the CFAI, CIM-C, and MSPSS-C scores after controlling for the FTSTS time and CESS scores. People experiencing energy-level deficiency will engage in adaptive behaviors to conserve energy, such as reducing participation in activities. Their community integration is also hindered. Social support appears to mediate fatigue, as it may allow stroke survivors to access help with their activities when they are fatigued.

### Clinical implications

In clinical practice, it is important to identify stroke survivors with fatigue and/or poor sleep quality because fatigue may affect their participation in rehabilitation exercises and thus delay their functional recovery. As sleep quality can predict fatigue following a stroke, it is plausible that interventions promoting sleep quality may alleviate fatigue. For example, a clinical trial of cognitive behavioral therapy was shown to improve both sleep quality and fatigue after a stroke [59]. Future studies are therefore warranted.

### Study limitations

This study has several limitations. First, the participants were self-selected from self-help groups, and were relatively socially active and mobile. This limited the representativeness of the sample. Second, being cross-sectional by design, this study could not explain the causal relationship between the variables. However, based on our findings, a longitudinal design could be used in future studies to develop a predictive model for fatigue following a stroke. Third, other factors, including depressive symptoms and comorbidities such as sleep-related breathing disorders, were not considered in this study. Their effects could be investigated in the future. Fourth, sleep quality was measured using a self-reported instrument. In future studies, accelerometers could be used to measure sleep efficiency. Last, detailed information about the use of sleep medications was not available in this study. Such data can be collected for analysis in future studies on fatigue and sleep quality.

## Conclusions

The prevalence rates of fatigue and poor sleep quality were found to be high in community-dwelling stroke survivors at ≥1 year after their last diagnosed stroke. Sleep quality independently predicted fatigue after a stroke and may be a target component in interventions to alleviate fatigue. Therefore, the level of fatigue and sleep quality should be assessed in clinical practice, such that prompt interventions can be provided.

## Data Availability

To maintain participant confidentiality, the datasets generated and/or analyzed during the current study are not publicly available, but can be obtained from the corresponding author on reasonable request.

## Abbreviations

CESS: Chinese version of the Epworth Sleepiness Scale
CFAI: Chinese version of the Frenchay Activities Index
C-FAS: Chinese version of the Fatigue Assessment Scale
CIM-C: Cantonese version of the Community Integration Measure
CLSA: Cantonese version of the Life-Space Assessment
CPSQI: Cantonese version of the Pittsburgh Sleep Quality Index
FMA-LE: Fugl-Meyer Assessment of the lower extremities
FMA-UE: Fugl-Meyer Assessment of the upper extremities
FTSTS: 5-Time Sit-To-Stand
ICC: Intraclass correlation coefficient
MSPSS-C: Chinese version of the Multidimensional Scale of Perceived Social Support
VAS-Pain: Visual Analogue Scale-Pain.
